# Severe Stenosis of Mitral Bioprosthetic Valve Thrombosis in a Patient with HCV-Related Cirrhosis and Duodenal Variceal Bleeding: The Deadly Triad

**DOI:** 10.3390/clinpract12050071

**Published:** 2022-08-29

**Authors:** Rosangela Cocchia, Salvatore Chianese, Giovanni Lombardi, Luigia Romano, Valentina Capone, Lucio Amitrano, Raffaele Bennato, Brigida Ranieri, Giuseppe Russo, Ciro Mauro, Eduardo Bossone

**Affiliations:** 1 Cardiology Unit, Antonio Cardarelli Hospital, 80131 Naples, Italy; 2 Gastroenterology Unit, Antonio Cardarelli Hospital, 80131 Naples, Italy; 3 Department of General and Emergency Radiology, Antonio Cardarelli Hospital, 80131 Naples, Italy; 4 Department of Advanced Biomedical Sciences, University of Naples Federico II, 80131 Naples, Italy; 5 IRCCS SYNLAB SDN, Via Emanuele Gianturco, 80143 Naples, Italy; 6 Chief Medical Officer, Antonio Cardarelli Hospital, 80131 Naples, Italy

**Keywords:** bioprosthetic mitral valve thrombosis, HCV-related cirrhosis, duodenal variceal bleeding

## Abstract

Bioprosthetic valve thrombosis (BPVT) is considered a relatively rare but life-threatening clinical entity. Thus, there is the need of high clinical suspicion in order to make a timely diagnosis and related appropriate therapeutic interventions. In this regard, the management of BPVT is high risk, whatever the option taken (surgery and/or systemic fibrinolysis). The presence of severe comorbidities—as decompensated cirrhosis—further complicates the clinical decision-making process, calling for a patient-tailored integrated multidisciplinary approach. We report a challenging case of a 45-year-old patient with mitral bioprosthetic valve thrombosis and hepatitis C virus (HCV)-related cirrhosis complicated by active duodenal variceal bleeding.

## 1. Introduction

Prosthetic valve thrombosis (PVT) represents a life-threatening cardiovascular emergency needing a prompt diagnosis and timely appropriate therapeutic intervention [1,2].

Portal hypertension is a severe complication of decompensated cirrhosis and is responsible for the development of ascites and bleeding from esophagogastric varices [3,4,5]. Approximately one-third of patients with histologically confirmed cirrhosis have varices, and one-third of patients with varices develop bleeding, with a mortality rate of approximately 40% [6,7,8].

We report a very challenging clinically unstable case of a 45-year-old patient with mitral bioprosthetic valve thrombosis (M-BPVT) and hepatitis C virus (HCV)-related cirrhosis complicated by duodenal variceal bleeding.

## 2. Case Description

A 45-year-old man, smoker, and former drug addict with known HCV-related cirrhosis (Child–Pugh C), was admitted to the emergency department for shortness of breath and melena. His vital signs were as follows: blood pressure 100/60 mmHg, heart rate 98/min, respiratory rate 19/minute, body temperature 37 °C, and peripheral oxygen saturation (SpO2) level 95%. Standard hematologic tests and blood chemistries showed severe anemia (hemoglobin 6.2 mg/dL), hepatic dysfunction (serum albumin 3.1 g/dL, AST 169 UI/L, 155 ALT UI/L, GGT 25 UI/L, bilirubin 1.5 mg/dL) including altered coagulation tests (INR 2.06, PT 39 %, aPTT 44.6 s). Impaired renal function was also observed (serum creatinine 4.93 mg/dL, eGFR 16 mL/min, KDIGO stage IV). Past medical history included recurrent variceal bleeding treated with cyanoacrylate injections, chronic coronary syndrome (occlusion of first and second diagonal branch on coronary angiography) and mitral valve infective endocarditis (*Staphylococcus aureus*) requiring mitral valve replacement surgery (St Jude Medical bio prosthesis 29). Subsequently, the M-BPV was complicated by major dehiscence and extensive “rocking motions” with related moderate paravalvular leak that was successfully treated with percutaneous intervention.

**Figure 1:**
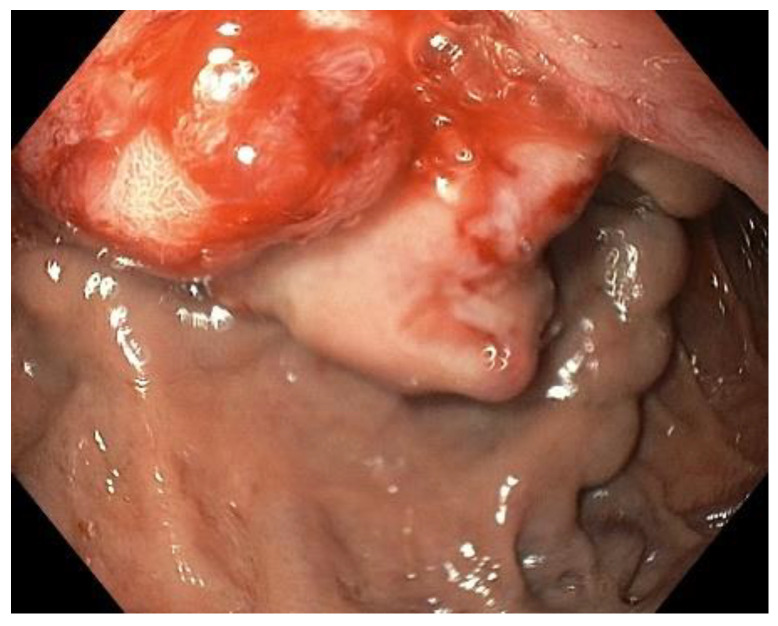
Esophago-gastro-duodenoscopy: duodenal variceal bleeding.

It should be highlighted that the patient had poor adherence to home therapy consisting of furosemide, spironolactone, low-dose aspirin, and ramipril. After the initial workup with transfusion of two packed red blood cells, the patient underwent esophago-gastro-duodenoscopy, which revealed duodenal variceal bleeding (Figure 1), which was subsequently treated with endoscopic injection sclerotherapy. He was started on medical therapy with combination of antibiotics, propranolol, furosemide, spironolactone, pantoprazole, lactulose, octreotide, and intravenous fluid with albumin.

**Figure 2:**
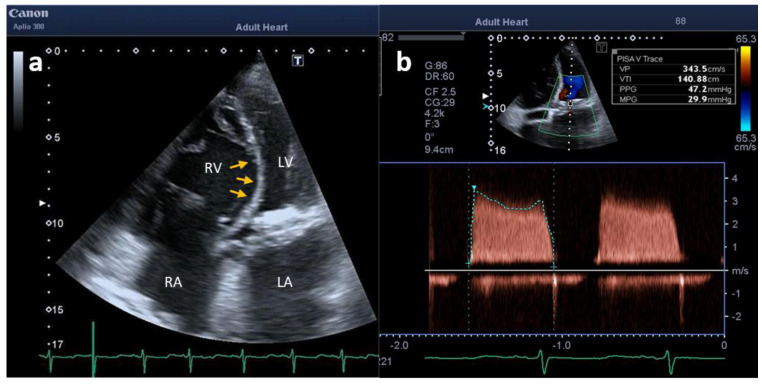
TTE showing (**a**) dilatation and overload of right chambers (arrows) in apical four − chamber view and (**b**) high trans—prosthetic mitral valve velocities and gradients (**b**). LA: left atrium; LV: left ventricle; RA: right atrium; RV: right ventricle.

**Figure 3:**
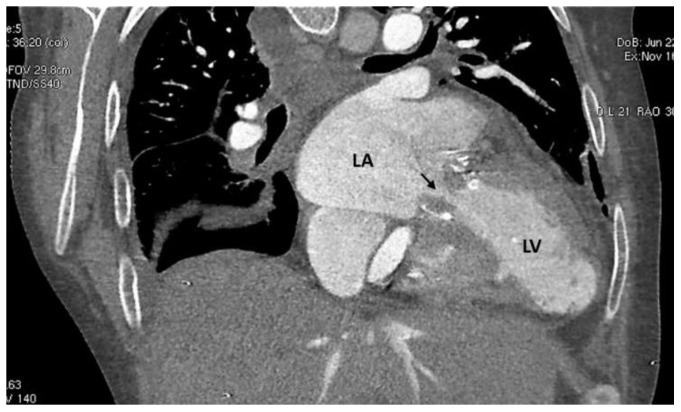
CCT showing obstruction to the blood flow across the stenotic mitral valve prosthesis (arrow). LA: left atrium; LV: left ventricle.

A bidimensional transthoracic Doppler echocardiography (TTE) exam was performed, which showed M-BPVT with severe stenosis (mean pressure gradient: 29.9 mmHg) and right ventricle dilatation/dysfunction along with severe pulmonary hypertension (estimated systolic pulmonary artery pressure: 70 mmHg) (Figure 2). A cardiac computed tomography (CCT) provided unique views of MVP dysfunction and excluded pulmonary embolism (Figure 3).

**Figure 4:**
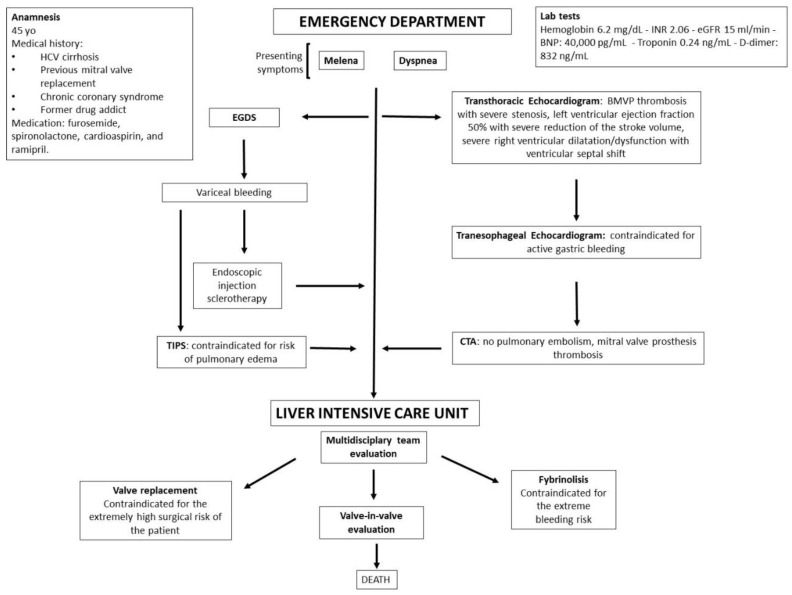
Timeline of events.

A transjugular intrahepatic portosystemic shunt (TIPS) was then excluded, as it could have precipitated the patient into pulmonary oedema as a direct consequence of volume overload [9]. For the worsening hemodynamic status, the patient was transferred to the liver intensive care where a multidisciplinary team was activated involving a cardiologist, cardiac surgeon, infectious disease specialist, pneumologist, and nephrologist (Figure 4). Different therapeutic interventions were evaluated: fibrinolysis was contraindicated for the extreme bleeding risk of the patient; valve replacement was excluded for the extremely high surgical risk of the procedure and valve-in-valve procedure was pending [10].

Eventually, the patient developed a septic shock and died of multiorgan failure.

## 3. Discussion

Obstructive PVT is a cardiovascular emergency with a high mortality rate (approximately 10%) independent of treatment modality [2].

Due to non-specific symptoms and signs, a high index of suspicion is warranted, and it should be suspected in patients with any type of prosthetic valve presenting with symptoms of heart failure, thromboembolism, and/or low cardiac output, new and pathologic heart murmurs, and/or documentation of inadequate anticoagulation [1,2]. The diagnosis should be rapidly confirmed by TTE and transesophageal (TOE) Doppler echocardiography (first-line imaging technique). Cinefluoroscopy, CCT, cardiac magnetic resonance imaging (CMR), and to a lesser extent nuclear imaging represent additional tools in the case of inconclusive echocardiography (TTE + TEE) and/or additional information is needed. (Table 1).

The management of a patient with M-BPVT and HCV-related cirrhosis complicated by duodenal variceal bleeding is high risk, whatever the option taken.

In general, the first therapeutic option to try in case of duodenal varicose veins is the endoscopic treatment. However, if the duodenal varices are large in size and/or located in the submucosal layer, there is a high risk of duodenal perforation, and it is difficult to access with an endoscope, so an intervention such as TIPS should be evaluated [17].

TIPS is known to be an effective and safe treatment for varicose bleeding, including duodenal varices, by lowering portal hypertension [17]. However, in this specific case, TIPS was limited by the high risk of pulmonary oedema as a direct consequence of volume overload [9].

A balloon-assisted retrograde transvenous obliteration (BRTO), or plug-assisted retrograde transvenous obliteration (PARTO), or coil-assisted retrograde transvenous obliteration (CARTO) could be performed (if specific expertise is present) as an alternative to TIPS depending upon the patient’s clinical status [18,19,20,21].

On the other hand, the M-BPVT treatment strategy depends on the patient’s hemodynamic status, the presence or absence of BPV obstruction, and valve location. Conventional treatment options include surgery, fibrinolysis, and anticoagulation [2]. Anticoagulation using a vitamin K antagonist (VKA) and/or UFH (unfractionated heparin) is the first-line treatment in hemodynamically stable M-BPVT [2].

For non-obstructive M-BPVT with large (>5 mm), mobile, and pedunculated thrombi, surgery could be considered if an intravenous heparin fails to resolve these features. For small (<5 mm) thrombi, medical therapy with oral anticoagulants (OAC) is usually the preferred option. In cases of obstructive thrombi, surgery or fibrinolysis could be considered [2].

Fibrinolysis may be considered if surgery is not an option or is very high risk for the treatment of thrombosis but carries a risk of bleeding and thromboembolism [2].

In this complex case, it was not possible to implement anticoagulation/systemic fibrinolysis for the high bleeding risk due to duodenal bleeding varices. Furthermore, it was not possible to undertake surgical mitral valve replacement due to the extremely high surgical risk of the patient [2,10,11,12] (Figure 4).

Finally, the patient had an unfortunate ending while a transcatheter valve-in-valve procedure evaluation was pending. However, the presence of a large thrombus could have led to high risk of periprocedural embolization [2].

In conclusion, in a challenging unstable clinical scenario such as the case presented here, it is of paramount importance to implement an integrated multidisciplinary team approach (involving a cardiologist, cardiac surgeon, internist, infectious disease specialist, hepatologist- gastroenterologist, pneumologist, and nephrologist) in order to evaluate the risk/benefit of every therapeutic intervention.

## Figures and Tables

**Table 1:** Mechanical vs. bioprosthetic valve thrombosis [2,10,11,12,13,14,15,16]. BPVT: bioprosthetic valve thrombosis; C-CMR: cardiac magnetic resonance imaging; CCT: cardiac computed tomography; OAC: oral anticoagulants; TOE: transesophageal echocardiography; TTE: trans-thoracic echocardiography; UFH: unfractionated heparin; VKA: vitamin k antagonist.

	Mechanical Prosthetic Valve Thrombosis	Bioprosthetic Valve Thrombosis
**Incidence in** **developed** **countries**	0.3% to 1.3%	0.03% to 0.5%
**Etiology**	Inadequate VKA anticoagulation (most frequent) Prosthesis malpositioning Hypercoagulable states	Inadequate antiplatelet or anticoagulant therapy Prosthesis malpositioning Hypercoagulable states
**Clinical presentation**	Stenotic murmur and muffled closing clicks Recent onset dyspnea Acute heart failure/cardiogenic shock Embolic event	Stenotic murmur Recent onset dyspnea Acute heart failure/cardiogenic shock. Embolic event
**Imaging**	TTE + TOE (1st line) If inconclusive echocardiography and/or additional information needed: Cinefluoroscopy CCT CMR *	TTE + TOE (1st line) If inconclusive echocardiography and/or additional information needed: Cinefluoroscopy CCT CMR *
**Main treatment**	**Obstructive Thrombosis** Urgent or emergency valve replacement is recommended for obstructive thrombosis in critically ill patients without serious comorbidity Fibrinolysis should be considered when surgery is not available or is very high risk, or for thrombosis of right-sided prostheses **Non-obstructive thrombosis** Surgery should be considered for large (>10 mm) non-obstructive prosthetic thrombus complicated by embolism Optimize anticoagulation first in other cases	*Anticoagulation using a VKA and/or UFH is recommended in BPVT s before considering re-intervention.* **Obstructive Thrombosis** Surgery in obstructed left-sided BPVT, hemodynamic instability, or decompensated heart failure. Fibrinolysis should be considered for high-risk surgical candidates and obstructive BPVT **Non-obstructive thrombosis** For non-obstructive left-sided BPVT with large (>5 mm), mobile, and pedunculated thrombi, surgery could be considered if intravenous heparin fails to resolve these features For small (<5 mm) thrombi, medical therapy with OAC is usually the preferred option

* Practically, all prosthetic heart valves, mechanical or bio-prosthetic, are considered safe in the MR environment at field strengths of up to 1.5 T. This is also true for most prosthetic valves at a higher field strength of 3 T, although evaluation is still pending for some valves.

## Data Availability

The data that support the findings of this case report are openly available in the References section.
